# Supplementary material for: Medial thalamic stroke and its impact on familiarity and recollection
Source: eLife. 2017 Aug 24;6:e28141. doi: 10.7554/eLife.28141 (PMC5595429; doi:10.7554/eLife.28141)
Supplement: Supplementary file 2. — The normalized volumes of the lesions are expressed in mm3. The extent of the lesions within the main thalamic nucleus groups (medial, lateral, anterior, posterior), subgroups (mediodorsal, intralaminar, midline) and individual nuclei (magnocellular MD, MDpc) is expressed as a percentage of volume loss according to Morel’s atlas. MTT volume loss is expressed as a percentage, according to Morel’s atlas. MTT volume is expressed as a z score compared with control participants. [file elife-28141-supp2.doc]

| **Group** | | | **iMTT** | | | | | | | **dMTT** | | | | | | | | |
| --- | --- | --- | --- | --- | --- | --- | --- | --- | --- | --- | --- | --- | --- | --- | --- | --- | --- | --- |
| **Participant** | | | P2 | P3 | P5 | P7 | P9 | Mean iMTT | SD iMTT | P1 | P4 | P6 | P8 | P10 | P11 | P12 | Mean dMTT | SD dMTT |
| **Overall lesion volume (manual segmentation, normalized mm^3^)** | | | 263 | 220 | 30 | 327 | 605 | 289 | 208.5 | 538 | 584 | 662 | 982 | 609 | 571 | 811 | 679.6 | 160.7 |
| **Lesions outside the thalamus (normalized %)** | | | 0 | 59 | 0 | 1.1 | 3.8 | 12.8 | 25.9 | 68.4 | 4.1 | 0 | 3 | 25.2 | 0 | 0 | 14.4 | 25.5 |
| **Morel atlas labels** | Medial group | MD | 11.5 | 0.2 | 0 | 9.8 | 29.7 | 10.3 | 12.1 | 18.2 | 2.4 | 25.2 | 20.7 | 0.2 | 20.3 | 19.9 | 15.3 | 9.8 |
| **(% of damage)** |  | MDmc | 1.9 | 0 | 0 | 2.9 | 20.3 | 5 | 8.6 | 7.3 | 0 | 18.8 | 28.5 | 0 | 24.6 | 20.8 | 14.3 | 11.8 |
|  |  | MDpc | 13.4 | 0.3 | 0 | 11.2 | 31.6 | 11.3 | 12.9 | 20.4 | 2.9 | 26.5 | 19.1 | 0.2 | 19.4 | 19.7 | 15.5 | 9.9 |
|  | Mean intra-laminar | | 13 | 17.3 | 2.3 | 25.8 | 14.3 | 14.5 | 8.5 | 19.3 | 0.9 | 2.4 | 3.1 | 0.3 | 1.6 | 2.8 | 4.4 | 6.7 |
|  | Mean midline | | 4.3 | 0.2 | 0 | 15.8 | 12.6 | 6.6 | 7.2 | 1.5 | 10.2 | 33 | 87.2 | 7.1 | 27 | 43.3 | 29.9 | 29.4 |
|  | Mean lateral group | | 1.6 | 0 | 0 | 2.8 | 2.6 | 1.4 | 1.4 | 0 | 27.6 | 9.4 | 23.1 | 19.3 | 8.5 | 12.8 | 14.4 | 9.5 |
|  | Anterior/posterior groups | | 0 | 0 | 0 | 0 | 0 | 0 | 0 | 4.9 | 2 | 0 | 0 | 0 | 0 | 0 | 0.3 | 0.8 |
|  | MTT | | 0 | 0 | 0 | 0 | 0 | 0 | 0 | 0 | 33.3 | 12.3 | 15.8 | 15.8 | 10.5 | 8.8 | 13.8 | 10.2 |
| **MTT volume (manual segmentation, Z score)** | | | -0.1 | -0.2 | -0.8 | 0.4 | -0.1 | -0.2 | 0.4 | -3.2 | -2.8 | -3.7 | -3.0 | -2.9 | -3.2 | -3.4 | -3.2 | 0.3 |
