# Supplementary material for: Medial thalamic stroke and its impact on familiarity and recollection
Source: eLife. 2017 Aug 24;6:e28141. doi: 10.7554/eLife.28141 (PMC5595429; doi:10.7554/eLife.28141)
Supplement: Transparent reporting form [file elife-28141-transrepform.docx]

***eLife’s* transparent reporting form**

We encourage authors to provide detailed information *within their submission* to facilitate the interpretation and replication of experiments. If you have any questions, please contact us: [editorial@elifesciences.org](mailto:editorial@elifesciences.org).

**Sample-size estimation**

- You should state whether an appropriate sample size was computed when the study was being designed
- You should state the statistical method of sample size computation and any required assumptions
- If no explicit power analysis was used, you should describe how you decided what sample (replicate) size (number) to use

Please outline where this information can be found within the submission (e.g., page numbers or figure legends), or explain why this information doesn’t apply to your submission:

We didn’t use any power analysis. Instead, we recruited patients with pure left thalamic strokes, which are particularly rare patients, until we had the largest group ever reported on recognition memory tasks to our knowledge.

**Replicates**

- You should report how often each experiment was performed
- You should include a definition of biological versus technical replication
- The data obtained should be provided and sufficient information should be provided to indicate the number of independent biological and/or technical replicates
- If you encountered any outliers, you should describe how these were handled
- Criteria for exclusion/inclusion of data should be clearly stated
- High-throughput sequence data should be uploaded before submission, with a private link for reviewers provided (these are available from both GEO and ArrayExpress)

Please outline where this information can be found within the submission (e.g., page numbers or figure legends), or explain why this information doesn’t apply to your submission:

- Each patient underwent each experiment only once.

- We haven’t removed any patients or control subjects for being outliers. However, two patients were removed from our initial group, as clearly stated in the methods section: “*We excluded one patient because of a depressive syndrome that impacted cognition, and one patient because a lacunar lesion was only visible on the T2 sequence in the acute phase*”.

- We haven’t excluded any data before any analyses. Data for one neuropsychological test is missing for one patient and for one behavioral scale for another patient, because they got tired (clearly indicated in Table 2). No experimental data (related to one of the three recognition memory task) is missing.

**Statistical reporting**

- Statistical analysis methods should be described and justified
- Raw data should be presented in figures whenever informative to do so (typically when N per group is less than 10)
- For each experiment, you should identify the statistical tests used, exact values of N, definitions of center, methods of multiple test correction, and dispersion and precision measures (e.g., mean, median, SD, SEM, confidence intervals; and, for the major substantive results, a measure of effect size (e.g., Pearson's r, Cohen's d)
- Report exact p-values wherever possible alongside the summary statistics and 95% confidence intervals. These should be reported for all key questions and not only when the p-value is less than 0.05.

- Statistical analyses methods are detailed at the end of the method section pages 16-17 and are reported as necessary throughout the result section pages 5-8.

- We provided the distribution of individual data for all figures where groups were compared (Figure 1, 2, 4). We also overlaid boxplots representing the minimum and maximum performance, the 25the and 75^th^ percentile as well as the median of the performance on Figures 1 and 4. Box plots whose notches do not overlap have different medians at the 5% significance level based on a normal distribution assumption. Outliers ( i.e., subjects whose performance fall outside minimum or maximum values of 1.5 the difference between the 25th and 75th percentile) would be represented by circles outside the minimum and maximum values.

Please outline where this information can be found within the submission (e.g., page numbers or figure legends), or explain why this information doesn’t apply to your submission:

(For large datasets, or papers with a very large number of statistical tests, you may upload a single table file with tests, Ns, etc., with reference to page numbers in the manuscript.)

**Additional data files (“source data”)**

- We encourage you to upload relevant additional data files, such as numerical data that are represented as a graph in a figure, or as a summary table
- Where provided, these should be in the most useful format, and they can be uploaded as “Source data” files linked to a main figure or table
- Include model definition files including the full list of parameters used
- Include code used for data analysis (e.g., R, MatLab)
- Avoid stating that data files are “available upon request”

Please indicate the figures or tables for which source data files have been provided:

We provide details of the data for Figure 4 in Supplementary file 1.

We provide details of the data for Figures 2 and 5, and for Table 2 in Supplementary file 2.
